# A Systematic Review Protocol of the Barriers to Both Physical Activity and Obesity Counselling in the Secondary Care Setting as Reported by Healthcare Providers

**DOI:** 10.3390/ijerph17041195

**Published:** 2020-02-13

**Authors:** Jaishri Sooknarine-Rajpatty, Austin B. Auyeung, Frank Doyle

**Affiliations:** Department of Health Psychology, Division of Population Health Sciences, Royal College of Surgeons in Ireland, 123 St. Stephen’s Green, D02 HX65 Dublin 2, Ireland; austinauyeung@rcsi.ie (A.B.A.); fdoyle4@rcsi.ie (F.D.)

**Keywords:** physical activity, obesity, counselling, barriers, systematic review

## Abstract

Physical activity and obesity counselling have both been gaining increasing interest in preventive health and treatment. However, most healthcare professionals do not provide advice on these topics nearly as often as they should. There are many reasons for this. Common barriers for the provision of brief advice on physical activity and obesity in both primary and secondary care are lack of time, motivation and knowledge. Systematic reviews have been published on the barriers of physical activity and obesity counselling in the primary care setting, but there is no published work on the barriers present in secondary care. This systematic review aims to assess all published data that discuss the barriers of physical activity and obesity counselling as noted by healthcare providers in secondary care. Four databases will be searched using the same search strategy, and the findings will be compiled using the COM-B model to explore the frequency of a reported barrier. This systematic review will be beneficial not only to practicing healthcare providers, but also the educational and managerial staff of secondary care facilities, as it may highlight the need for further training to fill gaps in the provision of preventive healthcare.

## 1. Introduction

Physical activity is a topic that constantly arises when a person’s health is discussed. It is a known fact that physical activity has many health benefits no matter a person’s age, gender, race, ethnicity, or disability, or if a person is pregnant or has a chronic illness [1]. According to the findings of a number of systematic reviews, which were conducted by the committee for the Physical Activity Guidelines for Americans, every adult (age 18 to 64) should be doing 150 to 300 min of moderate intensity, or 75 to 150 min of vigorous intensity aerobic activity per week, to receive significant health benefits [1]. An alternative combination of moderate and vigorous intensity aerobic activity can be utilized as long as the quota is achieved. For additional health benefits, muscle-strengthening activities of moderate or higher intensity that work all of the major muscle groups should be carried out on two or more days of the week [1]. Reviews produced by Swift et al. in the years 2014 and 2018 concluded that physical activity and exercise on their own do not consistently result in clinically significant weight loss or weight maintenance without a calorie-reduced diet [2,3]. However, there are numerous benefits to physical activity other than weight loss including improvement in mental health (treatment and prevention of dementia, reduced risk of future depressive disorder, decreased anxiety and increased cognition), reduced risk of cardiovascular disease, increased cardiorespiratory fitness in those with chronic illnesses, and improved circulation [4,5]. Statistics obtained from the U.S. Department of Health and Human Services stated that more than 80% of adults do not participate in the recommended amount of physical activity per week [6]. A 2006 narrative review by Warburton et al. appraised current publications at the time that discussed the significant role physical activity played in the prevention of chronic diseases (cardiovascular disease, cancer, diabetes, obesity, etc.) and premature death [7]. One major chronic illness that should be discussed is obesity, as it is a risk factor that eventually leads to the development of many other serious comorbidities, including hypertension, Type 2 Diabetes Mellitus, sleep apnoea, depression, all-cause mortality and many more [8].

Therefore, the provision of physical activity (PA) and obesity counselling by healthcare providers has become increasingly important. PA intervention should include a conversation about possible physical, mental and social barriers that may prevent the patient from partaking in physical activity, and the development of an individualized physical activity program in order to increase patient compliance and encourage a change in behaviour [9]. As long as these points are discussed, PA counselling does not require more than five minutes of a physician’s time. A patient is more likely to accept the advice of their physician and change their behaviour, as they believe physicians to be a credible source of preventive health information [10,11]. A healthcare provider who wishes to counsel their patient effectively on obesity should start by having an open discussion on general health and the many serious risks associated with being overweight or obese [12]. The healthcare provider should then assess whether the patient is willing to have a conversation about their weight and the possibility of making alterations to their diet and exercise regime. A 2012 systematic review and meta-analysis of published literature was performed by Rose et al., and it concluded that physician advice on obesity positively influences patients in their endeavor to lose weight [13].

However, there are many existing barriers to the provision of brief advice or counselling on physical activity and obesity by healthcare providers. A 2012 systematic review conducted by Hébert et al. identified the top three barriers to PA intervention in the primary care setting as lack of time, lack of knowledge or training in PA counselling and lack of perceived success in changing patient behaviour [14]. Frequent barriers to obesity counselling in the primary care setting, as determined by a pilot study undertaken by Forman-Hoffman et al. in 2006, were identified as lack of time; lack of training, resources and infrastructure; and fear that broaching the topic of obesity may cause friction in the doctor-patient relationship [15]. Börjesson (2013) stated that PA promotion in the hospital or secondary care setting may be effective to establish physical activity as the main treatment plan for lifestyle related risk factors and disorders [16]. The main obstacles, which may prevent physicians in secondary care facilities from educating their patients on physical activity, are a lack of motivation to give advice on what they believe to be preventive measures, lack of knowledge that PA has the potential to be the singular or additional treatment for a disease, lack of education and lack of structural support [16]. A scoping review carried out by Pearce in 2019 aimed to identify the role of the hospital and community health services in adult obesity prevention, as well as the potential enablers and barriers to the delivery of preventive health services. The barriers to obesity counselling discussed in this review included healthcare professional’s belief that their role does not address preventive health, and a lack of knowledge and confidence in broaching the topic of weight due to social conventions [17].

At present, there are a number of systematic reviews that focus on the barriers of PA and obesity counselling in the primary care setting. However, a systematic review that details the barriers of PA and obesity counselling in the secondary care setting as reported by healthcare professionals has yet to be conducted. Therefore, this systematic review aims to appraise all published literature that explores the potential barriers to brief advice or counselling on PA and obesity in the hospital or secondary care setting, and specific barriers will be grouped according to the COM-B model to allow readers to see how often each barrier has been reported [18]. The COM-B model of behaviour groups behaviours into three categories: capability, opportunity and motivation. This allows for a more organized and efficient approach when considering barriers of PA and obesity counselling and how this knowledge can be applied in a secondary care setting.

Objectives: This systematic review’s primary goal is to identify the major barriers of both physical activity and obesity counselling in the hospital and other secondary care facilities such as community-based programmes, social care services, palliative care services, nursing and retirement homes, specialist clinics, and rehabilitation services (excluding primary care). The participants will be all healthcare professionals, the intervention will be brief advice or counselling, there will be no comparators and the outcome will be barriers that have been noted by the healthcare providers. The barriers will be grouped using the COM-B model of behaviour (capability, opportunity and motivation), allowing readers to easily identify how many times a particular barrier has been reported [19]. The protocol will be conducted according to the PRISMA-P protocol (see Appendix A) [20]. As seen in the PRISMA-P 2015 statement, it is recommended that systematic review protocols be published as this ensures that the plans for a systematic review are documented and encourages consistent conduct by the review team, accountability and the integrity and transparency of the final systematic review. It also reduces bias associated with selective reporting of outcomes, as readers are able to recognize inconsistencies from the planned methods in the finished manuscript [20].

## 2. Materials and Methods

Registration: This systematic review protocol was registered with PROSPERO. The protocol number is forthcoming.

Eligibility Criteria: The participants and interventions are all healthcare professionals offering brief advice or counselling, respectively. The healthcare providers who will be included are physicians, surgeons, nurses, physiotherapists, pharmacists, social workers, nutritionists and dieticians. Healthcare professionals’ perceived barriers to PA and obesity counselling are the main outcomes of interest. Comparators are not relevant to this systematic review. There are no restrictions on the study type. Relevant settings may include hospitals, specialised clinics, and any other secondary care facilities. Publications based in the primary care setting will be excluded. Publications not written in English or that do not offer full-text access will be excluded from this systematic review. Relevant papers published prior to January 2020 will be included in this review.

Information Sources: The electronic databases to be searched are OVID MEDLINE, OVID PsychINFO, CINAHL and PubMed. A search will be performed in January 2020, and all publications relevant to this review will be included up until this date.

Search Strategy: The online databases OVID MEDLINE, OVID PsychINFO, CINAHL and PubMed will be searched for qualitative and quantitative publications using a predetermined designated search strategy. No limits will be placed on the search strategy, including date, language or study type. Appendix A shows the search strategy using OVID MEDLINE (see Appendix B), and the same strategy will be applied using the other three databases in January 2020.

### 2.1. Study Records

Data management: The results of the final search will be compiled in an EndNote library and combined with the final searches from the other three databases (OVID PsychINFO, CINAHL and PubMed).

Selection process: Two reviewers will independently screen the articles and select those that meet the inclusion criteria. Duplicates will be removed. Studies that do not have an English title or are based in the primary care setting will be excluded. The abstracts of the remaining articles will be reviewed to determine relevance.

Data collection process: A full-text copy of each research paper will be obtained. Two authors will review each paper independently and note specific barriers that have been identified. Any conflicting ideas or opinions will be resolved among the authors and, if necessary, a third reviewer.

Data items: The items included in this systematic review are perceived barriers to physical activity and obesity counselling in the hospital setting and specialised healthcare facilities, as reported by healthcare professionals.

Outcomes and prioritization: Outcomes will be prioritized based on the number of times a specific barrier has been noted. The findings will be grouped according to the COM-B model of behaviour (capability, opportunity and motivation).

Risk of bias in individual studies: One author will utilize the QualSyst tool to evaluate the risk of bias in all selected articles [21]. QualSyst has two manual checklists, one for quantitative studies with fourteen items and one for qualitative studies with ten items, to measure the risk of bias [21]. The author will record the percentage of checklist items met to simplify the process, with a higher percentage implying a lower risk of bias. Sensitivity analyses will consider results overall, but while also omitting studies with high risk of bias.

### 2.2. Data

Synthesis: Due to the diversity of study designs, the authors will compile a narrative of the data obtained from the research papers. Qualitative and quantitative studies will be analysed separately and conclusions drawn. The authors will synthesize data on the barriers of physical activity and obesity counselling in the hospital and secondary care setting. These barriers will be categorized according to the COM-B model of behaviour in order to identify how frequently each barrier has been recorded. Due to significant heterogeneity of the studies, a narrative, qualitative summary will be generated in the form of text and tables, allowing the findings from the studies to be conceptualized. An additional table will be provided taking into account the sample size, the types of healthcare professionals involved, and the barriers that were reported by the professionals in each included study. This will allow readers to appreciate the size of each study and the healthcare providers that reported certain barriers.

## 3. Discussion

This will be the first systematic review to gather all of the barriers to the provision of PA and obesity counselling as detailed by healthcare providers in secondary care settings. The barriers that are noted will be arranged as per the COM-B model, and will be discussed in further detail. The results of this systematic review will be useful, as it will not only educate practicing healthcare providers but will also inform education and managerial staff. The foundation for more refined training programs in secondary healthcare facilities could assist in making healthcare professionals more capable when addressing PA and obesity. A more focused approach to PA and obesity counselling will improve healthcare professional’s delivery and efficacy, potentially translating to improved patient health outcomes.

## 4. Conclusion

Similar to previous research in the provision of brief smoking cessation advice [19], this systematic review will be beneficial not only to practicing healthcare providers, but also the educational and managerial staff of secondary care facilities, as it may highlight the need for further training to fill gaps in the provision of preventive healthcare.

## Appendix A

**Table A1:** PRISMA P Checklist 2015 for Systematic Review Protocols [20].

Section/Topic	Item Number(s)	Checklist Item	Information Reported		Line Number(s)
			Yes	No	
**ADMINISTRATIVE INFORMATION**					
**Title**					
Identification	1a	Identify the report as a protocol of a systematic review			
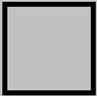
Update	1b	If the protocol is for an update of a previous systematic review, identify as such			
Registration	2	If registered, provide the name of the registry (e.g., PROSPERO) and registration number in the Abstract			
**Authors**					
Contact	3a	Provide name, institutional affiliation and e-mail address of all protocol authors; provide physical mailing address of corresponding author			
Contributions	3b	Describe contributions of protocol authors and identify the guarantor of the review			
Amendments	4	If the protocol represents an amendment of a previously completed or published protocol, identify as such and list changes; otherwise, state plan for documenting important protocol amendments			
**Support**					
Sources	5a	Indicate sources of financial or other support for the review			
Sponsor	5b	Provide name for the review funder and/or sponsor			
Role of sponsor/funder	5c	Describe roles of funder(s), sponsor(s) and/or institution(s), if any, in developing the protocol			
**INTRODUCTION**					
Rationale	6	Describe the rationale for the review in the context of what is already known			
Objectives	7	Provide an explicit statement of the question(s) the review will address with reference to participants, interventions, comparators and outcomes (PICO)			
**METHODS**					
Eligibility criteria	8	Specify the study characteristics (e.g., PICO, study design, setting, time frame) and report characteristics (e.g., years considered, language, publication status) to be used as criteria for eligibility for the review			
Information sources	9	Describe all intended information sources (e.g., electronic databases, contact with study authors, trial registers, or other grey literature sources) with planned dates of coverage			
Search strategy	10	Present draft of search strategy to be used for at least one electronic database, including planned limits, such that it could be repeated			
***STUDY RECORDS***					
Data management	11a	Describe the mechanism(s) that will be used to manage records and data throughout the review			
Selection process	11b	State the process that will be used for selecting studies (e.g., two independent reviewers) through each phase of the review (i.e., screening, eligibility, and inclusion in meta-analysis)			
Data collection process	11c	Describe planned method of extracting data from reports (e.g., piloting forms, done independently, in duplicate), and any processes for obtaining and confirming data from investigators			
Data items	12	List and define all variables for which data will be sought (e.g., PICO items, funding sources), and any pre-planned data assumptions and simplifications			
Outcomes and prioritization	13	List and define all outcomes for which data will be sought, including prioritization of main and additional outcomes, with a rationale			
Risk of bias in individual studies	14	Describe anticipated methods for assessing risk of bias of individual studies, including whether this will be done at the outcome or study level, or both; state how this information will be used in data synthesis			
***DATA***					
Synthesis	15a	Describe criteria under which study data will be quantitatively synthesized			
	15b	If data are appropriate for quantitative synthesis, describe planned summary measures, methods of handling data, and methods of combining data from studies, including any planned exploration of consistency (e.g., *I* ^2^, Kendall’s tau)			
	15c	Describe any proposed additional analyses (e.g., sensitivity or subgroup analyses, meta-regression)			
	15d	If quantitative synthesis is not appropriate, describe the type of summary planned			
Meta-bias(es)	16	Specify any planned assessment of meta-bias(es) (e.g., publication bias across studies, selective reporting within studies)			
Confidence in cumulative evidence	17	Describe how the strength of the body of evidence will be assessed (e.g., GRADE)			

## Appendix B

**Table A2:** OVID MEDLINE Search Strategy.

Search Number	Query
S1	(MH Physicians+)
S2	(TI Physician * OR AB physician*) OR (TI Clinician* OR AB Clinician *) OR (TI Doctor* OR AB Doctor *) OR (TI Surgeon * OR AB Surgeon *) OR (TI Practitioner * OR AB Practitioner *) OR (TI Professional * OR AB Professional) OR (TI Health Professional * OR AB Health Professional *) OR (TI Professional Role * OR AB Professional Role *)
S3	(TI Nurse * OR AB Nurse *) OR (TI Nursing * OR AB Nursing *) OR (TI Nurses Role * OR AB Nurse Role *)
S4	(TI Physiotherapist * OR AB Physiotherapist *) OR (TI Physical therapist * OR AB Physical Therapist *) OR (TI Chiropractor * OR AB Chiropractor *) OR (TI Massage therapist * OR AB Massage therapist *) OR (TI Occupational therapist * OR AB Occupational therapist *) OR (TI Rehabilitation therapist * OR AB Rehabilitation therapist *) OR (TI Acupuncturist * OR AB Acupuncturist *) OR (TI Kinesiologist * OR AB Kinesiologist *) OR (TI Pharmacist * OR AB Pharmacist *) OR (TI Social worker * OR AB Social worker *) OR (TI Nutritionist * OR AB Nutritionist *) OR (TI Dietician * OR AB Dietician *)
S5	(MH Hospitals+)
S6	(TI Hospital * OR AB Hospital *) OR (TI Health Facilities * OR AB Health Facilities *) OR (TI Patient * OR AB Patient *) OR (TI Inpatient * OR AB Inpatient *) OR (TI Emergency Department * OR AB Emergency Department *) OR (TI Emergency Room * OR AB Emergency Room *) OR (TI Ward * OR AB Ward *) OR (TI Secondary Care * OR AB Secondary Care *) OR (TI Community healthcare * OR AB Community Healthcare *) OR (TI Specialist clinic * OR AB Specialist clinic *) OR (TI Social care * OR AB Social care *) OR (TI Rehabilitation centre * OR AB Rehabilitation centre *) OR (TI Palliative care * OR AB Palliative care *) OR (TI Nursing home * OR AB Nursing home *) OR (TI Retirement home * OR AB Retirement home *) OR (TI Tertiary Care * OR AB Tertiary Care *)
S7	(MH Exercise+)
S8	(TI Inactivity * OR AB Inactivity *) OR (TI Physically inactive * OR AB Physically Inactive *) OR (TI Sedentary Lifestyle * OR AB Sedentary Lifestyle *) OR (TI Obese * OR AB Obese *) OR (TI Obesity * OR AB Obesity *) OR (TI Overweight * OR AB Overweight *) OR (TI Overnutrition * OR AB Overnutrition *) OR (TI Body Weight * OR AB Body Weight *) OR (TI Body Size * OR AB Body Size *) OR (TI Body Composition * OR AB Body Composition *) OR (TI Body Mass Index * OR AB Body Mass Index *) OR (TI Adiposity * OR AB Adiposity *) OR (TI Adipose Tissue * OR AB Adipose Tissue *) OR (TI Waist Circumference * OR AB Waist Circumference *)
S9	(TI Weight Loss * OR AB Weight Loss *) OR (TI Physical Activity * OR AB Physical Activity *) OR (TI Exercise Education * OR AB Exercise Education *) OR (TI Exercise Program * OR AB Exercise Program *) OR (TI Exercise Benefits * OR AB Exercise Benefits *) OR (TI Health Promotion * OR AB Health Promotion *)
S10	(TI Intervention * OR AB Intervention *) OR (TI Minimal Intervention * OR AB Minimal Intervention *) OR (TI Brief Intervention * OR AB Brief Intervention *) OR (TI Brief Advice * OR AB Brief Advice *) OR (TI Delivery of Healthcare * OR AB Delivery of Healthcare *) OR (TI Counselling * OR AB Counselling *) OR (TI Obesity Counselling * OR AB Obesity Counselling *) OR (TI Directive Counselling * OR AB Directive Counselling *) OR (TI Training * OR AB Training *) OR (TI Interview * OR AB Interview *) OR (TI Motivational Interviewing * OR AB Motivational Interviewing *) OR (TI Screening * OR AB Screening *)
S11	(TI Barrier * OR AB Barrier *) OR (TI Factor * OR AB Factor *) OR (TI Challenge * OR AB Challenge *) OR (TI Obstacles * OR Obstacles *) OR (TI Attitudes * OR AB Attitudes *) OR (TI Attitude of Health Personnel * OR AB Attitude of Health Personnel *) OR (TI Perceived * OR AB Perceived *) OR (TI Perception * OR AB Perception *) OR (TI Opinion * OR AB Opinion *) OR (TI Belief * OR AB Belief *) OR (TI Knowledge * OR AB Knowledge *) OR (TI Implementation * OR Ab Implementation *) OR (TI Facilitator * OR AB Facilitator *)
S12	S1 OR S2 OR S3 OR S4
S13	S5 OR S6
S14	S12 AND S13
S15	S7 OR S8 OR S9
S16	S10 AND S11 AND S14 AND S15

* This is used as a wildcard symbol to widen the search for words starting with the same letters.
